# Syphilitic Affections of the Heart and Aorta

**Published:** 1903-09-19

**Authors:** 


### SYPHILITIC AFFECTIONS OF THE HEART AND AORTA.

Syphilis has been perhaps rather neglected by medical practitioners in general as a cause of lesions occurring in the heart and great vessels, although the connection has been sufficiently remarked upon by pathologists. In calling attention to this question Dr. Weber[1] states that, although not common, syphilis of the heart does occur and that a proper recognition of this fact is important from the point of view both of prognosis and treatment, for antisyphilitic treatment will arrest the degenerative process. As to the appearances met with at the autopsy of a syphilitic heart or aorta, Dr. Weber affirms that, apart from gumma in the cardiac muscle or cardiac arteries, there is nothing in the appearance of the sclerotic changes of the vessels or in the softening and degeneration of the muscular fibres that would enable it to be said with certainty whether these conditions were due to syphilis or to other causes. The commonest lesions are atheromatous patches in the aorta and pulmonary and coronary arteries and, as seen under the microscope, endarteritis and periarteritis. In other cases the heart will show areas of softening in the myocardium with replacement of the muscular fibres by fibrous tissue. The diagnosis, in the absence of syphilitic stigmata, may be almost impossible at an early stage. But the signs of myocarditis such as weak cardiac impulse with accentuated second sound, arythmic action, tachycardia, faintness, dyspnœa on exertion, angina pectoris, general depression and weakness, occurring in individuals below fifty years of age should always arouse a suspicion of syphilis, whether the patient admits a previous infection or denies it. The effect of specific treatment may clear up the diagnosis in a doubtful case. To rely upon the iodides alone in the treatment of cardiac syphilis will, according to Dr. Weber's experience, lead to disappointment; temporary improvement will be obtained, perhaps, but it will soon pass away. Not so if mercury be given in addition, and this Dr. Weber prefers to administer by inunction whenever practicable.

[1] Medical Record, Aug. 29, 1903.

